# Modification of Bacterial Effector Proteins Inside Eukaryotic Host Cells

**DOI:** 10.3389/fcimb.2016.00073

**Published:** 2016-07-20

**Authors:** Crina M. Popa, Mitsuaki Tabuchi, Marc Valls

**Affiliations:** ^1^Department of Genetics, Centre for Research in Agricultural Genomics (CSIC-IRTA-UAB), Universitat de BarcelonaBarcelona, Spain; ^2^Department of Applied Biological Science, Faculty of Agriculture, Kagawa UniversityKagawa, Japan

**Keywords:** type III secretion system, type IV secretion system, bacterial effector, bacterial virulence, eukaryotic host, animal pathogens, plant pathogens

## Abstract

Pathogenic bacteria manipulate their hosts by delivering a number of virulence proteins -called effectors- directly into the plant or animal cells. Recent findings have shown that such effectors can suffer covalent modifications inside the eukaryotic cells. Here, we summarize the recent reports where effector modifications by the eukaryotic machinery have been described. We restrict our focus on proteins secreted by the type III or type IV systems, excluding other bacterial toxins. We describe the known examples of effectors whose enzymatic activity is triggered by interaction with plant and animal cell factors, including GTPases, E2-Ubiquitin conjugates, cyclophilin and thioredoxins. We focus on the structural interactions with these factors and their influence on effector function. We also review the described examples of host-mediated post-translational effector modifications which are required for proper subcellular location and function. These host-specific covalent modifications include phosphorylation, ubiquitination, SUMOylation, and lipidations such as prenylation, fatty acylation and phospholipid binding.

## Activation of bacterial effectors through interaction with host factors

Many effectors from bacterial pathogens of both animals and plants contain catalytic domains on their primary sequences with predicted enzymatic activities, such as phospholipase, protease, protein kinase, transferase, etc. and some of them have been found to be highly active enzymes that can outcompete their eukaryotic counterparts (Levin et al., 2010). However, not all effectors exhibit the enzyme activity when expressed in bacterial systems, but rather require interaction with additional eukaryotic factors for activation. Recent findings provide remarkable examples of spatiotemporal regulation of bacterial effectors by coupling the catalytic activity to the arrival into a host cell cytoplasm (Table 1; Anderson et al., 2015). Here, we will describe the available examples of the structure-based activation of effectors from animal and plant bacterial pathogens through interaction with host cell factors.

**Table 1:** **Bacterial effectors modified by host factors ordered by species**.

**Organism**	**Effector**	**Modification**	**Host eukaryotic factor**	**Effector activity**	**Process/Target(s)**	**References**
*Bartonella henselae*	BepD, BepE, BepF	Phosphorylation	Src family Tyr kinases	Unknown	Actin cytoskeleton(?)/MAPK signaling(?)	Schulein et al., 2005; Selbach et al., 2009
*Chlamydia trachomatis*	AmpA	SUMOylation	SUMO1 (?), SUMO2/3	Unknown	Unknown	Beyer et al., 2015
*Chlamydia trachomatis*	AnkA	Phosphorylation	Tyr kinases Src, Abl-1	Unknown	SHP-1/Histone deacetylase 1/Chromatin remodeling	Jw et al., 2007; Lin et al., 2007; Rennoll-Bankert et al., 2015
*Chlamydia trachomatis*	Tarp	Phosphorylation	Src family kinases, Abl, Syk	Putative paxillin-like activity	Actin cytoskeleton/MAPK signaling	Clifton et al., 2004; Mehlitz et al., 2010; Thwaites et al., 2014
*Chlamydia trachomatis*	TepP	Phosphorylation	Unknown kinases	Unknown	Crk signaling	Chen et al., 2014
*Entero-pathogenic Escherichia coli*	Tir	Phosphorylation	PKA, Tyr kinases Src, Fyn, Abl	Unknown	Actin cytoskeleton	Phillips et al., 2004; Brandt et al., 2009; Selbach et al., 2009
*Helicobacter pylori*	CagA	Phosphorylation	Tyr kinase Src, Abl	Unknown	Actin cytoskeleton/MAPK signaling	Segal et al., 1999; Backert and Selbach, 2005; Selbach et al., 2009; Backert et al., 2010b
*Legionella pneumophila*	AnkB	Ubiquitination/Prenylation - farnesylation	Unknown enzymes, Trim21 (?)/Ras, Rab, Rho family(?)	F-box protein	Trim21, SCF1 complex	Price et al., 2010a; Bruckert and Abu Kwaik, 2015
*Legionella pneumophila*	GobX, LpdA	Palmitoylation	Unknown	E3 ubiquitin ligase	Unknown	Lin et al., 2015; Schroeder et al., 2015
*Legionella pneumophila*	PelA, PelH,	Multiple prenylation	Unknown farnesyl and geranylgeranyl-tranferases	Remodeling of *Legionella*-containing vacuole	Unknown	Ivanov et al., 2010
*Legionella pneumophila*	PelE, PelF, PelJ	Prenylation	Ras, Rab, Rho family(?)	Evasion of lysosomal fusion	Unknown	Price et al., 2010b
*Legionella pneumophila*	SetA, SidC, SidM, LidA	Phospholipid binding	Unknown	Glycosyltransferase, Ub ligase, adenylyl-transferase	Vesicular trafficking, Rab GTPases/Phosphoinositide	Haneburger and Hilbi, 2013; Ivanov and Roy, 2013
*Legionella pneumophila*	VipD	Activation	Rab5	Phospholipase A1	Phospholipids (PI3P)	Gaspar and Machner, 2014
*Pseudomonas aeruginosa*	ExoS	Activation	14-3-3	GAP/ADP-ribosyl-transferase	Rho/Rac/Cdc42	Fu et al., 1993
*Pseudomonas aeruginosa*	ExoT	Activation	14-3-3	GAP/ADP-ribosyl-transferase	Rho/Rac/Cdc42	Fu et al., 1993
*Pseudomonas aeruginosa*	ExoU	Activation/Phospholipid binding	Ubiquitin	Phospholipase A2	Phospholipids	Anderson et al., 2011; Gendrin et al., 2012
*Pseudomonas syringae*	AvrB	Activation/Phosphorylation/Myristoylation	Unknown kinases	Unknown	RIN4	Nimchuk et al., 2000; Desveaux et al., 2007
*Pseudomonas syringae*	AvrPphB, ORF4	Myristoylation/Palmitoylation	Unknown	Cysteine protease	Unknown	Dowen et al., 2009
*Pseudomonas syringae*	AvrPto	Phosphorylation/Myristoylation/Palmitoylation	Unknown kinases	Unknown	FLS2, EFR	Shan et al., 2000; Thara et al., 2004; Anderson et al., 2006
*Pseudomonas syringae*	AvrPtoB	Phosphorylation/Ubiquitination	Pto kinase/ unknown kinases/ UbcH5a, UbcH5c, UbcH6	E3 ubiquitin ligase	Fen, CERK1, FLS2, BAK1, Ubiquitin	Abramovitch et al., 2006; Janjusevic et al., 2006; Xiao et al., 2007; Ntoukakis et al., 2009; Mathieu et al., 2014
*Pseudomonas syringae*	AvrRpm1	Myristoylation/Palmitoylation	Unknown	Suppression of plant defense responses	Unknown	Nimchuk et al., 2000
*Pseudomonas syringae*	AvrRpt2	Activation	Cyclophilin	Cystein protease	RIN4	Axtell et al., 2003; Coaker et al., 2005
*Pseudomonas syringae*	HopF2, HopZ1a, HopZ1b, HopZ1c, HopZ2, HopZ4	Myristoylation	IP6	ADP-ribosyl-transferase/acetyltransferase+ unknown functions	RIN4/JAZ proteins, tubulin…	He et al., 2006; Robert-Seilaniantz et al., 2006; Lewis et al., 2008; Lee et al., 2012; Üstün et al., 2014
*Pseudomonas syringae*	HopQ1	Phosphorylation	Unknown kinases	Unknown	14-3-3 proteins	Li et al., 2013
*Ralstonia solanacearum*	RipAY	Activation	Thio-redoxin	γ-glutamyl cyclotransferase	Glutathione, unknown γ-glutamyl compounds	Fujiwara et al., 2016
*Rhizobium sp*.	NopL, NopP	Phosphorylation	Unknown/MAP kinases/PKA	Unknown	MAPK pathways (?)	Bartsev et al., 2003; Skorpil et al., 2005; Zhang et al., 2011
*Salmonella typhimurium*	SifA	Prenylation–geranylgeranyl addition/S-acylation	Ras, Rab, Rho family/geranylgeranyl transferase I	Putative Rho GTPase	Rho1p	Reinicke et al., 2005
*Salmonella typhimurium*	SopA	Ubiquitination	HsRMA, UbcH5a, UbcH5c, UbcH7	E3 ubiquitin ligase	Unknown	Zhang et al., 2005, 2006
*Salmonella typhimurium*	SopB/SigD	Ubiquitination	TRAF6, UbcH5c	Phosphoinositide phosphatase	Actin/Phosphoinositide/Cdc42	Marcus et al., 2002; Rogers et al., 2008; Knodler et al., 2009; Patel et al., 2009; Ruan et al., 2014
*Salmonella typhimurium*	SopE, SptP	Ubiquitination	Unknown enzymes	Guanine nucleotide exchange factor, GTPase activating protein	Rac1, Cdc42	Kubori and Galan, 2003
*Salmonella typhimurium*	SseJ	Activation	RhoA	Glycero-phospholipid-cholesterol acetyltransferase (GCAT)	Cholesterol	Christen et al., 2009
*Salmonella typhimurium*	SspH2, SseI	Palmitoylation	Unknown palmitoyl-transferases	E3 ubiquitin ligase	Nod1, IQGAP1	Hicks et al., 2011; Ivanov and Roy, 2013
*Shigella spp*.	OspG	Activation/Ubiquitination	E2~ubiquitin	Ser/Thr kinase	NFκB signaling pathway	Zhou et al., 2013; Pruneda et al., 2014
*Sinorhizobium fredii*	NopT	Myristoylation/Palmitoylation	Unknown	YopT-like cysteine protease	Unknown	Dowen et al., 2009
*Xanthomonas campestris pv. campestris*	XopE1, XopE2, XopJ, AvrXccC	Myristoylation	Unknown	Ser/Thr acetyltransferase, cysteine protease	RPT6, unknown	Thieme et al., 2007; Wang et al., 2007; Üstün et al., 2013; Üstün and Börnke, 2015
*Xanthomonas campestris pv. vesicatoria*	AvrBsT	Phosphorylation	PIK1	Putative YopJ-like Ser/Thr acetyltransferase	SGT1 (cell division) signaling	Kim et al., 2014
*Yersinia enterocolitica*	YopE	Ubiquitination	Unknown enzymes	GTPase activating protein	Actin cytoskeleton/Rac1, RhoA, Cdc42	Ruckdeschel et al., 2006; Hentschke et al., 2007
*Yersinia spp*.	YopJ	Activation	IP6	Acetyl- transferase	MEK	Mittal et al., 2010
*Yersinia spp*.	YpkA/YopO	Activation	G-actin	Ser/Thr kinase	Actin-regulating proteins	Juris et al., 2000

### Allosteric activation of *Legionella* effector VipD by host GTPase Rab5

**Figure 1:**
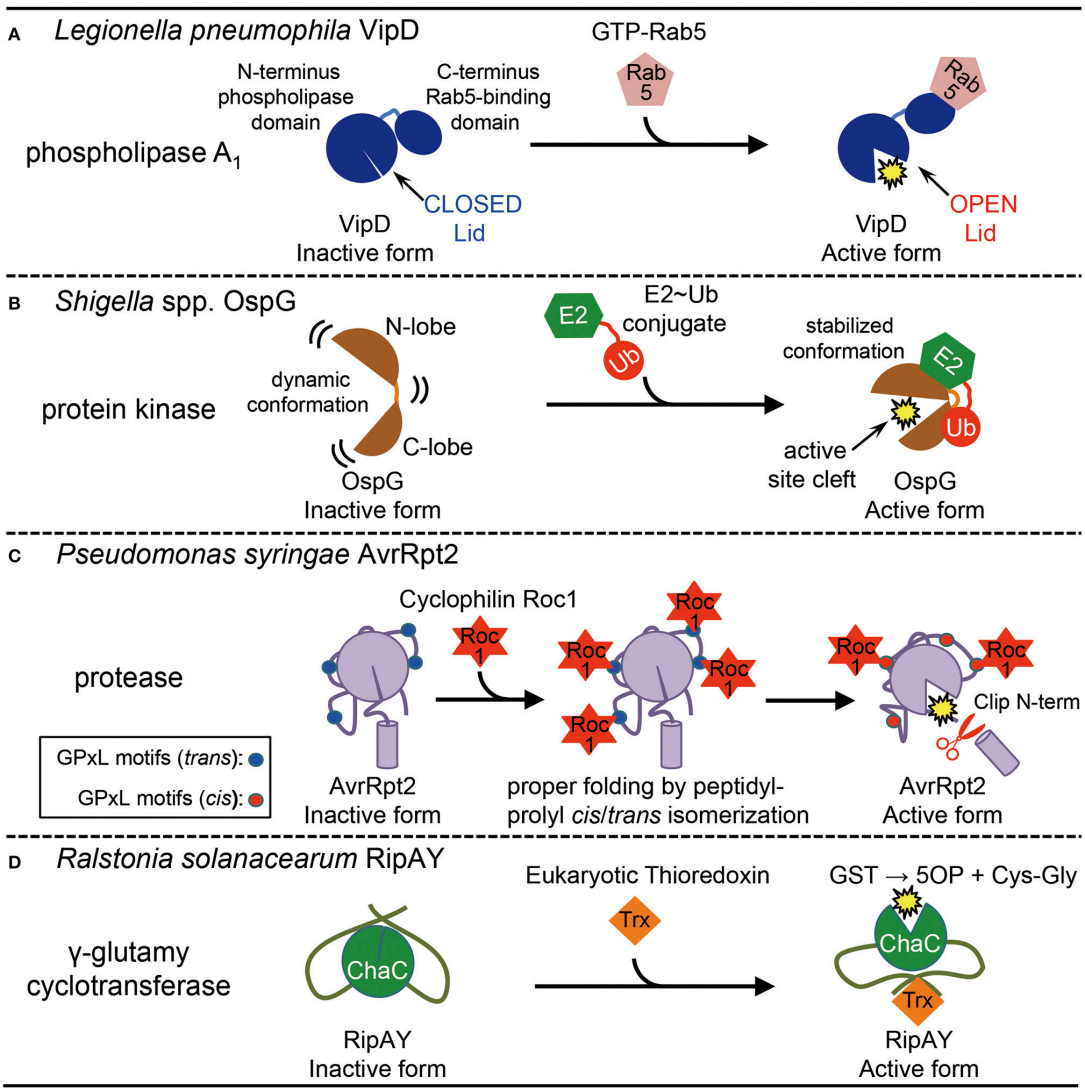
**Activation of bacterial effector proteins by host eukaryotic factors. (A)** Activation of *Legionella pneumophila* effector VipD by host small GTPase Rab5. Rab5-binding induces conformational change to open the lid, which results in the activation of its phospholipase A1 activity. **(B)** Activation of *Shigella* spp. effector OspG by host E2-ubiquitin (Ub) conjugate. E2~Ub binding stabilizes an active site cleft of the kinase, which stimulates enzymatic activity. **(C)** Activation of Pseudomonas syringae effector AvrRpt2 by host cyclophilin Roc1. Roc1 binds AvrRpt2 at the four potential cyclophilin-binding sites (GPxL) and properly folds the protein by cis/trans peptidyl-prolyl isomerization, which stimulates the protease activity. **(D)** Activation of *Ralstonia solanacearum* effector RipAY by host eukaryotic thioredoxins. Binding of eukaryotic thioredoxins stimulates the γ-glutamyl cyclotransferase activity of ChaC domain in RipAY by unknown mechanisms. GST, glutathione; 5OP, 5-oxoproline; Cys-Gly, cysteinylglycine.

Upon uptake by macrophages, *Legionella pneumophila*, the causative agent of legionnaires' disease (Horwitz and Silverstein, 1980), injects more than 250 effector proteins through the Dot/Icm type IV secretion system (T4SS) into the host cell. These effectors allow escape from the phagosomal maturation and establishment of a *Legionella*-containing vacuole (LCV) that supports bacterial proliferation (Ensminger and Isberg, 2009). The effector VipD can remove the endosomal specific phospholipid, phosphaptidyinositol-3 phosphate, PI(3)P by its robust phospholipase A_1_ (PLA_1_) activity, which is stimulated by the host GTPase Rab5, a key regulator of endosomes (Gaspar and Machner, 2014). Depletion of PI(3)P by VipD causes membrane disassociation of the endosomal fusion proteins including the tethering protein early endosomal antigen (EEA)1, resulting in inhibition of phagosomal maturation and allowing endosomal avoidance by LCVs. The N-terminal half of VipD possesses high homology to patatin, a lipid acyl hydrolase present in the potato tuber, whereas the C-terminal half of VipD is required for binding to the Rab5 to trigger PLA_1_ activity within the N-terminal domain (Gaspar and Machner, 2014). The crystal structure of VipD confirmed the predicted bimodular organization and in addition, revealed a surface loop called “lid,” that obstructs a PLA_1_ active site (Figure 1A, Closed Lid), explaining why recombinant VipD alone exhibits little or no PLA_1_ activity *in vitro* (Ku et al., 2012). The crystal structure of VipD in complex with constitutively active Rab5 provides evidence for a heterotropic allosteric activation mechanism in which locally induced structural changes through Rab5-binding are transmitted from the C-terminal domain of VipD to the N-terminal PLA_1_ domain, causing the reposition of the lid and exposure of the catalytic pocket (Figure 1A, Open Lid; Lucas et al., 2014).

### E2-Ub conjugates stabilize an active conformation of *Shigella* effector OspG

*Shigella* spp. are human pathogens that cause shigellosis and utilize a type III secretion system (T3SS) to deliver over 20 effector proteins to hijack cellular processes of the host and promote bacterial invasion, survival, and proliferation (Buchrieser et al., 2000). The *Shigella* effector OspG represents a minimal kinase domain that retains key catalytic elements, but lacks additional structural features typically found in eukaryotic kinases (Kim et al., 2005). Using a yeast two-hybrid analysis and pull-down experiments, Kim et al. (2005) identified that OspG binds to the ubiquitin (Ub)-conjugating enzymes (E2s) covalently linked with Ub (E2~Ub) and exhibits weak kinase activity *in vitro* (Kim et al., 2005). The crystal structure of OspG in complex with E2~Ub conjugate uncovered how E2~Ub conjugate-binding stimulates the kinase activity of OspG (Grishin et al., 2014; Pruneda et al., 2014). While OspG alone appears to be highly dynamic and weakly active, complex formation stabilizes a highly active conformation via simultaneous interaction with both subunits of the E2~Ub conjugate (Figure 1B). *In vitro* kinase assay revealed that OspG alone exhibits very weak kinase activity, while OspG in complex with E2~Ub conjugate exhibits substantially greater activity. Consistent with a model in which the OspG kinase domain is stabilized by binding to an intact E2~Ub conjugate, the covalent linkage between E2 enzyme and Ub is required for maximal activation of the kinase *in vitro*. It was recently showen that OspG also binds ubiquitin and polyubiqutin chains and this binding stimulates its kinase activity (see below).

### Eukaryotic cyclophilin-dependent conformational change activates *P. syringae* effector AvrRpt2

The plant bacterial pathogen, *Pseudomonas syringae* injects between 20 and 30 effector proteins into host plant cells via the T3SS (Chang et al., 2005). Delivery of the effector AvrRpt2 to *Arabidopsis thaliana* plants expressing the plant resistance protein RPS2 specifically induces a hypersensitive response leading to disease resistance (Day et al., 2005). AvrRpt2 possesses cysteine protease activity and cleaves the Arabidopsis protein RIN4, which negatively regulates resistance interacting with RPS2 (Axtell et al., 2003). RPS2 is activated following RIN4 cleavage, thereby indirectly detecting AvrRpt2's enzymatic activity (Axtell and Staskawicz, 2003; Mackey et al., 2003). Interestingly, AvrRpt2 is delivered into plant cells as an inactive protease that is activated *in planta* and autoprocessed to trigger RIN4 degradation and subsequent activation of RPS2. Coaker et al. (2005), demonstrated that plant cyclophilin triggers both self-cleavage of AvrRpt2 and limited degradation of RIN4 (Coaker et al., 2005). Cyclophilin possesses peptidyl-prolyl *cis*/*trans* isomerase activity, which facilitates protein folding catalyzing the *trans* to *cis* isomerization of peptide bonds at proline residues (Kiefhaber et al., 1990). Enzymes from *Arabidopsis* plant deficient in peptidyl-prolyl *cis*/*trans* isomerization were unable to activate AvrRpt2 *in vitro*, indicating that this activity is key for AvrRpt2 activation (Coaker et al., 2006). Interestingly, AvrRpt2 possesses four consensus cyclophilin-binding motifs, GPxLs which are located in close proximity to ArRpt2's catalytic triad and are required for enzymatic activity both *in vitro* and *in planta*. Nuclear magnetic resonance spectra and gel filtration chromatography suggest that AvrRpt2 may only be structured and active when ROC1 or another cyclophilins is bound in order to maintain one or more proline residues in the appropriate isomerization state (Figure 1C; Coaker et al., 2006), which is also supported by analysis of protease activity using synthetic protease substrate (Aumüller et al., 2010).

### Activation of *R. solanacearum* effector RipAY by host thioredoxins

*R. solanacearum* is a widely distributed soil borne phytopathogen that possesses an exceptionally large (60–75) T3SS effector repertoire, for which only a few members have been assigned a molecular function (Coll and Valls, 2013). RipAY was identified as one of the few *Ralstonia* effectors causing growth inhibition in yeast (Fujiwara et al., 2016). RipAY contains a ChaC domain, which is conserved in all phyla and recently shown to encode γ-glutamyl cyclotransferase (GGCT) activity specifically to degrade glutathione (Kumar et al., 2012, 2015). RipAY has N- and C-terminal extension sequences outside of its ChaC domain, so that is much larger than other ChaC proteins (416 amino acids vs ~200 amino). In spite of the limited identity of its ChaC domain to the consensus sequence, RipAY was found to exhibit a robust GGCT activity in yeast cells. In addition, intracellular glutathione levels were significantly decreased following *ripAY* expression in yeast or inoculation of *R. solanacearum* wild-type, but not a RipAY-deficient strain, into plant leaves. Recombinant RipAY protein purified from a bacterial expression system showed undetectable GGCT activity, but addition of eukaryotic thioredoxins (Trxs) stimulated this activity *in vitro* (Figure 1D; Fujiwara et al., 2016). Yeast two hybrid analysis revealed that RipAY bound to plant cytoplasmic thioredoxins in an isoform-specific manner. RipAY preferentially bond to the plant cytoplasmic thioredoxin Trx-h5, whose expression is specifically induced during pathogen infection and the GGCT activity of RipAY was most efficiently stimulated by Trx-h5. Unlike the requirement of cyclophilin enzymatic activity for AvrRpt2 activation, the Trx-h5 oxide reductase activity is not indispensable, although a Trx-h5 redox inactive mutant showed decreased binding and activation of RipAY. The crystal structure of RipAY in complex with thioredoxin will uncover the mechanisms underlying recognition and activation of RipAY at the molecular level.

## Post-translational modification of pathogen effectors inside the host cells

Besides interacting with the host components, bacterial effectors can suffer a number of different post-translational modifications in the eukaryotic environment. These host-specific covalent modifications include phosphorylation, ubiquitination, SUMOylation and lipidation—mainly prenylation and fatty acylation. The main function of these modifications seems to be effector targeting to a precise subcellular compartment or regulation of its biological activity

### Serine and threonine phosphorylation of plant-associated type III effectors

Specific kinases attach negatively charged phosphate groups to the phosphorylatable aminoacid residues (histidine, serine, threonine, and tyrosine) of their substrates, which regulates their function and changes their biochemical properties (Backert and Selbach, 2005; Korkuc and Walther, 2016). Such post-translational modifications by the host are essential for some plant-associated virulence proteins to manipulate host defense signaling. It is the case of *Pseudomonas syringae* type III effectors AvrPto and AvrPtoB, known to elicit plant resistance in the form of a hypersensitive cell death (Coll et al., 2011) after interaction with the tomato immunity-associated kinase Pto (Kim et al., 2002). Phosphorylation on two serine residues of AvrPto C-terminal domain was shown to contribute to both its virulence and avirulence activity inside the host cell (Anderson et al., 2006; Yeam et al., 2010). *P. syringae* strains carrying mutations in AvrPto S149, a phosphorylation site confirmed *in vivo*, and in S147 caused less severe disease symptoms in susceptible tomato plants lacking Pto kinase (Anderson et al., 2006). By contrast, these alterations still elicited cell death in resistant tomato cultivars, as a result of unaffected Pto-mediated recognition of AvrPto (Yeam et al., 2010). Still, phosphorylation on S147 and S149 was required for AvrPto recognition by a putative resistance protein in *Nicotiana sylvestris* and *Nicotiana tabacum* (Yeam et al., 2010). The Pto-independent kinase activity responsible for AvrPto phosphorylation was observed in various plant species (Anderson et al., 2006), however it seems that not all hosts use similar recognition mechanisms for the same effector.

Similarly, AvrPtoB was shown to be phosphorylated on two amino acid residues by host kinases (Xiao et al., 2007; Ntoukakis et al., 2009). Whereas phosphorylation on serine-258 contributed to AvrPtoB full activity (Xiao et al., 2007), phosphorylation on threonine-450 by Pto kinase itself had controversial outcomes on AvrPtoB-mediated recognition in resistant tomatoes. Ntoukakis and coworkers demonstrated that Pto kinase phosphorylates AvrPtoB on a threonine residue in its E3 ubiquitin ligase C-terminal domain, leading to effector inactivation and its inability to degrade the tomato kinase and suppress the immune response (Ntoukakis et al., 2009). On the contrary, another study described that Pto binding to the N-terminal domain of AvrPtoB (which includes residue serine-258), and not T450 phosphorylation, allows this kinase to evade degradation and activate immunity in response to the effector protein (Mathieu et al., 2014).

Other effectors from phytobacteria are substrates for host immunity kinases. Recent findings highlight phosphorylation as a key event during the resistance response of the host plant to the action of *Xanthomonas campestris* pv *vesicatoria* AvrBsT effector (Kim et al., 2014). Phosphorylation of AvrBsT by PIK1 (Pathogen-Induced Protein Kinase1), dependent on the presence of SGT1 (part of a protein complex with AvrBsT and PIK1), was shown to play a role in effector recognition and cell death-associated phenotype in *N. benthamiana* leaves (Kim et al., 2014). In a likewise manner, *P. syringae* HopQ1 phosphorylation on serine-51 residue strongly promoted bacterial virulence and modulated effector interaction with multiple tomato 14-3-3 proteins (Li et al., 2013). This is in accordance to a recent study, confirming a regulatory role of phosphorylation in compound binding (Korkuc and Walther, 2016).

Finally, two effectors from the symbiotic *Rhizobium* strain NGR234, NopL and NopP, are phosphorylated *in vitro* by different plant kinases, including MAPKs (Bartsev et al., 2003, 2004; Skorpil et al., 2005). Further experiments confirmed four phosphorylated serines of NopL and revealed this effector interference with MAPK pathways in yeast and tobacco, but it is yet unknown whether phosphorylation is involved in NopL and NopP function (Zhang et al., 2011).

### Tyrosine phosphorylation of effector proteins from animal-associated bacteria

Phosphoproteomic studies have demonstrated that phosphorylation of proteins on tyrosine residues occurs at a minor ratio compared to serine and threonine phosphorylation (Olsen et al., 2006). Nevertheless, tyrosine phosphorylation is crucial for the regulation of processes like growth, division and differentiation in all eukaryotes, and it has recently emerged as a key circuit controlling many cellular functions in bacteria (Hunter, 2009; Whitmore and Lamont, 2012). In the last decades, many effector proteins from animal-associated bacteria such as enteropathogenic *Escherichia coli, Helicobacter pylori, Chlamydia trachomatis, Bartonella henselae*, and *Anaplasma phagocytophilum* have been shown to target and perturb host tyrosine (Tyr) phosphorylation mechanisms. *H. pylori* effector CagA and Tir from *E. coli* possess Tyr phosphorylation sites within conserved Glu-Pro-Ile-Tyr-Ala or related sequence motifs, described to be modified by host cytosolic kinases involved in signal transduction, including Src, Abl, and Fyn (Phillips et al., 2004; Tegtmeyer and Backert, 2011). Time-dependent regulation of CagA tyrosine phosphorylation process by host Src and Abl family kinases (Mueller et al., 2012) plays a direct role in effector activation and triggering of host cell morphological changes related to cytoskeletal rearrangements and induction of cell elongation (Backert et al., 2010b; Sougleri et al., 2016). Similarly, manipulation of actin signaling by *E. coli* Tir requires phosphorylated tyrosine-454 and tyrosine-474 residues, suggesting these phosphorylation events are triggered by infection with the bacteria and are crucial for the effector activity inside host (Campellone and Leong, 2003; Bommarius et al., 2007). Furthermore, *E. coli* and wild-type Tir specifically activate host protein kinase A (PKA), which phosphorylates Tir at serine-434 and serine-463, modifications that, in contrast to CagA-induced phenotype, inhibit cell elongation (Brandt et al., 2009; Backert et al., 2010a). Recent studies concluded multiple phosphorylation sites of type III or type IV effectors like CagA (*H. pylori*), Tir (EPEC *E. coli*), BepD-F (*Bartonella henselae*), Tarp (*Chlamydia trachomatis*) and AnkA (*Anaplasma phagocytolium*) allow these bacterial proteins to interfere with host cellular signaling at different levels, by recruiting a rich repertoire of interacting partners (Selbach et al., 2009; Hayashi et al., 2013).

*Chlamydia trachomatis* Tarp is another effector shown to undergo tyrosine phosphorylation, immediately after its translocation inside the host cell (Clifton et al., 2004). In this case, Tarp phosphorylation was required and led to an increase in the number of effector interactions with host partners, such as the human adaptor protein SHC1, involved in activation of growth and MAPK signaling (Mehlitz et al., 2010). Recent findings identified a novel *Chlamydia* type III effector, TepP, whose interaction with Crk, another host adaptor protein, depends on effector phosphorylation at tyrosine and serine residues (Chen et al., 2014). Interestingly, tyrosine phosphorylation of TepP was shown to occur later than Tarp phosphorylation, suggesting that *C. trachomatis* together with a type III secretion chaperone Slc1 are able to regulate translocation of the effector repertoire to the pathogen's benefit (Chen et al., 2014).

### Ubiquitin-dependent function of *Pseudomonas syringae, Salmonella, Yersinia* and *Legionella* effector proteins

Ubiquitination is a post-translational protein modification involving the addition of the small (8.5 kDa) ubiquitin molecule on lysine residues (rarely on cysteine and serine) in the substrate N-terminus (Behrends and Harper, 2011). Attachment of a single ubiquitin moiety is called monoubiquitination, and it can affect the localization or the activity of the target protein (Ramanathan and Ye, 2012). The ubiquitin (Ub) subunit is itself modified on one or more of its seven lysine residues, leading to the formation of a poly-Ub chain (polyubiquitination), which can constitute a signal for target protein degradation or play a role in modulating substrate function (Behrends and Harper, 2011). Because ubiquitination regulates and participates in many cellular functions such as protein degradation, cell cycle, vesicle trafficking or immune responses, some bacterial effectors have evolved to exploit this system by binding to or modifying host ubiquitin components, while other effectors are themselves subjected to ubiquitination (Angot et al., 2007; Zhou and Zhu, 2015). *Yersinia* YopE and *Salmonella thyphimurium* SopA, SopB/SigD, SopE, and SptP type III effectors are all ubiquitinated after their translocation inside the host cell (Ruckdeschel et al., 2006; Narayanan and Edelmann, 2014). Time-dependent ubiquitination of SopE and SptP leaded to their degradation by the host ubiquitin-proteasome system (Kubori and Galan, 2003). Similarly, YopE belonging to *Y. enterocolitica* serotype O8, but not its homologs from serogroups O3 and O9, was shown to be polyubiquitinated at lysine-62 and lysine-72, suggesting that some effectors have evolved to escape ubiquitination and subsequent degradation by the host (Hentschke et al., 2007). This is also the case of type III effector SopB, whose host-mediated ubiquitination serves as a non-proteolytic signal and contributes to effector function and intracellular localization (Thomas and Holden, 2009; Narayanan and Edelmann, 2014). For example, a SopB mutant that cannot be ubiquitinated demonstrated that effector modification on any of its nine lysine residues is required for redistribution from the plasma membrane to the *Salmonella*-containing vacuole (SCV) and recruitment of the small GTPase Rab5 (Patel et al., 2009). Importantly, SopB forms -regardless of their ubiquitination status- are still present at the plasma membrane and function to stimulate bacterial internalization and actin remodeling. Effector delivery to SCV by the host-ubiquitin machinery concentrated SopB activities, such as alteration of phosphoinositide metabolism, at this site, which allows *Salmonella* to escape degradation by the lysosomes (Knodler et al., 2009; Patel et al., 2009). Regarding the host enzyme(s) that modify SopB, it was recently described that its ubiquitination is mediated by the E2 ubiquitin-conjugating UbcH5c enzyme and the TRAF6 member of E3 ubiquitin ligases (Ruan et al., 2014), enzymes regulating substrate specificity in the ubiquitination process (Berndsen and Wolberger, 2014). SopA is itself an ubiquitin E3 ligase, polyubiquitinated by the host HsRMA1, with the same E3 ligase activity (Zhang et al., 2005, 2006). Although SopA ubiquitination by HsRMA1 finally leads to effector “sacrifice” and its proteasomal degradation, it also serves as a signal regulating *Salmonella* escape into the cytosol, where it can rapidly multiply (Zhang et al., 2005). In a likewise manner, ubiquitination of *P. syringae* AvrPtoB by host enzymes, together with the effector intrinsic E3 ligase activity, play a role in AvrPtoB interaction with ubiquitin itself and suppression of plant immunity (Abramovitch et al., 2006; Janjusevic et al., 2006).

Finally, *Shigella* spp. OspG and *L. pneumophila* AnkB are also ubiquitinated. OspG binds ubiquitin and polyubiqutin chains, which stimulates its kinase activity (Zhou et al., 2013) and AnkB is polyubiquitinated through Lys11 (Bruckert and Abu Kwaik, 2015). Lys11-linked polyubiquitinated AnkB is not degraded by the proteasome, suggesting this post-translational effector modification might lead to other cellular outcomes, distinct from the established function of Lys11-linked chains as proteasomal targeting signals (Behrends and Harper, 2011; Bruckert and Abu Kwaik, 2015). Additional data would be needed to thoroughly understand the biological significance of the host-mediated polyubiquitination of this *Legionella* effector protein.

### AmpA, a bacterial SUMOylated effector

Functionally distinct from the ubiquitin pathway, protein SUMOylation involves target substrate modification of one or more lysine residues by covalent attachment of a member of the small ubiquitin-like modifier (SUMO) family of proteins (Guo and Henley, 2014). By altering interactions of the modified substrate, or changing its localization, stability and activity, SUMO conjugation controls a broad network of cellular processes, including nuclear processes, metabolic pathways, endocytic trafficking of receptors and resistance to pathogens (Wilson, 2012).

Many pathogenic bacteria were described to exploit and negatively regulate host SUMOylation system (Wilson, 2012; Verma et al., 2015), however there are few cases when pathogens utilize this essential pathway to “adorn” their own effectors. Effector protein AmpA (*Anaplasma phagocytophilum*) was shown to be poly-SUMOylated by conjugation to SUMO2/3 and this modification promoted bacterial survival inside the host (Beyer et al., 2015). Although the molecular consequences of AmpA host-mediated SUMOylation are yet unknown, Beyer and coworkers insinuate this modification would offer the possibility to manipulate a wide range of host activities to a bacterium with a limited number of effector proteins (Beyer et al., 2015).

### Prenylation of the effectors AnkB and SifA

S-prenylation covalently adds isoprene groups, usually farnesyl (15-carbon) and geranylgeranyl (20-carbon), to specific cysteine residues within 5 amino acids from a protein C-terminus via thioether linkages. The CaaX (Cys—aliphatic—aliphatic—X) motif is the most common prenylation site in proteins, a reaction carried out by farnesyl transferase, Caax protease and geranylgeranyl transferase I (Casey and Seabra, 1996). Prenyl moieties can play an important role in increasing molecular hydrophobicity, so that they serve as mediators of membrane association or determine specific protein-protein interaction (Ivanov and Roy, 2013). Unlike S-palmitoylation (see below), S-prenylation is an irreversible process.

PelH and AnkB from *Legionella pneumophila* are known examples of farnesylated bacterial effectors (Price et al., 2010a,b). Specific inhibitors and mutant cell lines showed that host-dependent farnesylation -but not geranylgeranylation- of AnkB was shown to be indispensable for its anchoring to the cytosolic face of the membrane surrounding the LCV. This modification was also shown to be essential for biological function, as bacteria bearing a mutation in the farnesylated cysteine showed a reduced capacity to proliferate in mice lungs (Price et al., 2010a). The same experiments performed with PelH demonstrated that its farnesylation is essential for proper membrane location (Price et al., 2010b).

Prenylation by geranylgeranyl addition has been indirectly proven for the *Salmonella typhimurium* effector protein SifA. SifA is required for maintenance of the membrane that surrounds replicating bacteria in the so-called SCV. It was shown that for SifA targeting and association to membranes a C-terminal cysteine in a conserved CAAX and Rab geranylgeranyl transferase prenylation motif was required (Reinicke et al., 2005). This cysteine residue within the CAAX was shown to be modified by isoprenoid addition through the action of protein geranylgeranyl transferase I (Reinicke et al., 2005).

Prenylation may be a conserved mechanism for effector modification in animal pathogens, as *in silico* analyses show that most bacterial species contain effectors with the conserved prenylation motif. An exhaustive *in silico* screen of microbial genomes for C-terminal CXXX-motif-containing proteins identified 56 proteins (Al-Quadan et al., 2011), 10 of them corresponding to *Legionella pneumophila* type IV effectors (Ivanov et al., 2010). Mutation of this motif in the Legionella effectors or inhibition of isoprenoid biosynthesis in the host cell confirmed lipidation of AnkB, and PelH, and showed altered membrane localization of PelA, PelE, PelJ, and PelF. Treatment with specific enzyme inhibitors showed that AnkB and PelJ are farnesylated, whereas PelE and PelF are modified by a geranylgeranyltransferase (Ivanov et al., 2010). Prenylated eukaryotic proteins include Ras and members of the Rab and Rho families. It will be interesting to check whether effectors mimicking these eukaryotic activities (Popa et al., 2016) are also prenylated.

### Effector fatty acylation: myristoylation in plant cells and palmitoylation in animal cells

Acylation involves the covalent attachment of fatty acids at certain amino acid residues. The saturated myristic (14-carbon) acid and palmitic acid (16-carbon) are the most common fatty acids covalently attached to proteins, providing different biochemical characteristics to the protein. Myristoylation is a common acylation through which a myristic acid is attached to the α-amino group of an N-terminal glycine residue through an amide linkage. This irreversible protein modification typically occurs co-translationally (Martin et al., 2011). Addition to a myristoyl group provides proteins sufficient hydrophobicity and affinity for membranes, but it is insufficient to maintain permanent association with them (Resh, 2006). For this reason, myristoylation is often combined with S-acylation on proximal cysteine residues (see below). S-palmitoylation is another acylation in which a palmitic acid is attached to the thiolate side chain of a cysteine residue via thioester linkage. In contrast to N-myristoylation, S-acylation is a post-translational and reversible modification and no consensus sequence for protein palmitoylation has been identified so far. Because of its long hydrophobic group, S-palmitoylation can permanently anchor the protein to the membrane, sometimes concentrated at lipid rafts and thioesterases can release the protein by cleaving the linkage to the lipid (Resh, 2006).

Myristoylation has been described in a number of effectors from bacterial plant pathogens. The *Pseudomonas syringae* effectors AvrRpm1 and AvrB were the first shown to require a consensus Glycine2 fatty acid acylation site for full functionality and to be myristoylated in the plant host cell (Nimchuk et al., 2000). This seems a common strategy for *P. syringae* effectors, as myristoylation sites in HopF2, AvrPphB, AvrPto, and four of the five HopZ family effectors (HopZ1a, HopZ1b, HopZ1c, and HopZ2) are also required for targeting these effectors to the plant plasma membrane (He et al., 2006; Robert-Seilaniantz et al., 2006; Lewis et al., 2008). The putative myristoylation site was essential for HopZ2 and HopF2 virulence functions and for HopZ1a and AvrPto recognition by the plant immune system. In the case of AvrPphB, the eukaryotic N-myristoylation site was only exposed after protein autoprocessing in the plant cell and direct binding of the lipid was proven (Nimchuk et al., 2000). In a recent report, HopZ4 was also shown to the plasma membrane and this location was required for its activity as a proteasome inhibitor (Üstün et al., 2014). The *Xanthomonas* effectors XopE1, XopE2, XopJ, and AvrXccC are also anchored to the plant plasma membrane via myristoylation, as point mutations in their putative myristoylated G2 glycine residues resulted in cytoplasmic localization (Thieme et al., 2007; Wang et al., 2007). In the case of XopJ -a cysteine protease that degrades its target RPT6 (Üstün and Börnke, 2015)- a G2A exchange in the N-terminal myristoylation motif also abolished its proteasome inhibitor activity inside host cells (Üstün et al., 2013). Although these results suggest that acylation plays an important role in effector function, direct myristoylation in plant host cells has not been demonstrated so far for any of them.

Contrary to mysristoylation, palmitoylation is more common in effectors from animal bacterial pathogens. For instance, the Salmonella effector proteins SspH2 and SseI are localized to the plasma membrane of host cells through S-palmitoylation of a conserved cysteine residue within their N-terminal domains. In these cases, lipidation is mediated by specific palmitoyltransferases from the host cell and is critical for effector function (Hicks et al., 2011; Ivanov and Roy, 2013). Interestingly, palmitoylated SspH2, and SseI are targeted to different domains of the plasma membrane, suggesting that this modification is not sufficient for proper localisation. Legionella effectors GobX and LpdA were also shown to be post-translationally modified by palmitoylation, which targets them to the Golgi or the Rab4- and Rab14-containing endosomes, respectively (Lin et al., 2015; Schroeder et al., 2015). AvrPto and AvrPphB from the plant pathogen *P. syringae* is an exception to the described specific acylation in animal vs plant cells, as they can be palmitoylated. For AvrPto disruption of a putative myristoylation motif abolished membrane association and its avirulence activity in tomato and tobacco. Regarding AvrPphB was shown be palmitoylated -as well as myristoylated- inside the plant cells (see below).

### Phospholipid binding to effectors in animal cells

Phosphoinositideipids are phosphorylated derivatives of phosphatidylinositol and control key cellular processes, such as vesicular trafficking. Different phosphoinositide species target different intracellular membranes, so that they can play an essential role as anchor moieties to target proteins to precise locations. *L. pneumophila* type IV effectors SetA, SidC, SidM, and LidA have been shown to bind different phosphoinositides to target the LCV (Haneburger and Hilbi, 2013). This lipid association and the ensuing location is essential to carry out their function promoting interaction of the LCV with the host organelles (Ivanov and Roy, 2013). The type III-secreted effector ExoU from *Pseudomonas aeruginosa* is also modified by phosphoinositide binding. ExoU acts as a phospholipase that is localized to the plasma membrane. Effector binding to precise phosphatidylinositol species that are abundant at the cytoplasmic side of the plasma membrane was shown to be required for its location and activity as a necrotic factor to promote bacterial multiplication (Gendrin et al., 2012).

### Multiple lipidation of effectors

Protein modification with palmitate can stably target to the plasma membrane proteins previously modified by other types of lipidation, such as myristoylation or farnesylation (Resh, 2006). It has been proposed that multiple protein lipidation would start with N-myristoylation, which would target the protein to the endomembrane system, followed by S-acylation—normally S-palmitoylation of nearby cysteines-, which enhances membrane association of lipidated proteins (Resh, 1999). This process also takes place for some effectors once in their eukaryotic target cells. The first report of multiple lipidation was *Pseudomonas syringae* effector AvrRpm1, in which a G2A mutation in the myristoylation site eliminated membrane localization, while a C3A exchange in the putative palmitoylation site reduced membrane association (Nimchuk et al., 2000). This is logical given the requirement for myristoylation to occur before palmitoylation. In this same work, palmitoylation consensus sequences were identified in AvrRpm1, AvrB, AvrC, AvrPto, and AvrPphB. Some years later, multiple lipidation by both myristoylation and palmitoylation were demonstrated by heterologous expression in yeast for the *Pseudomonas syringae* effectors AvrPphB and ORF4 and their related effector NopT from *Sinorhizobium fredii* (Dowen et al., 2009). As described for AvrPphB, these effectors are auto-processed inside the plant cell, exposing the previously hidden acylation motifs. Lipidation targeted the effectors to the plasma membrane, which was required, at least for AvrPphB, to exert its functions. Interestingly, myristoylation-deficient variants of these effectors were also not palmitoylated, indicating that the former modification is required for subsequent acylation (Dowen et al., 2009). Although not proven biochemically, conservation of the predicted dual acylation motif containing G2 and a proximal cysteine suggests that at least 10 more *Pseudomonas* and *Xanthomonas* effectors may be modified in plant cells by myristoylation and S-acylation (Hicks and Galán, 2013).

The *S. typhimurium* effector protein SifA was shown to be modified both by the animal host cell prenylation (see above) and the S-acylation machineries (Reinicke et al., 2005). Interestingly, mutation of the S-acylation motif in SifA did not affect bacterial survival in the host, whereas disruption of the prenylated residue attenuated bacterial growth in the rat liver, suggesting the latter modification plays a more prominent role in bacterial virulence (Reinicke et al., 2005).

Finally, various forms of prenylation have also been proposed to coexist in a single effector. For instance, location of the *L. pneumophila* type IV effectors PelA and PelH is dependent on prenylation but neither farnesyltransferase nor geranylgeranyltransferase inhibitors perturbed their localization, suggesting that both enzymes can modify them (Ivanov et al., 2010).

## Author contributions

CP wrote the manuscript. MT wrote the manuscript. MV coordinated manuscript preparation and wrote the manuscript.

## Funding

This work was funded by projects AGL2013-46898-R (MINECO, Spain) to MV and COST Action FA1208 Sustain funded by the European Union H2020. This work was also supported by Japan Society of the Promotion of Science KAKENHI Grant 16K07668.

### Conflict of interest statement

The authors declare that the research was conducted in the absence of any commercial or financial relationships that could be construed as a potential conflict of interest.
